# Insights into the relationship between usability and willingness to use a robot in the future workplaces: Studying the mediating role of trust and the moderating roles of age and STARA

**DOI:** 10.1371/journal.pone.0268942

**Published:** 2022-06-03

**Authors:** Mohammad Babamiri, Rashid Heidarimoghadam, Fakhradin Ghasemi, Leili Tapak, Alireza Mortezapour

**Affiliations:** 1 Department of Ergonomics, Health Sciences Research Center, School of Public Health, Hamadan University of Medical Sciences, Hamadan, Iran; 2 Occupational Health and Safety Engineering Department, Abadan University of Medical Sciences, Abadan, Iran; 3 Department of Biostatistics, School of Public of Health, Hamadan University of Medical Sciences, Hamadan, Iran; 4 Department of Ergonomics, School of Public Health, Hamadan University of Medical Sciences, Hamadan, Iran; Universitat de Valencia, SPAIN

## Abstract

**Background and aim:**

Human–robot collaboration is the key component of the fourth industrial revolution concept. Workers’ willingness to collaborate with industrial robots is a basic requirement for an efficient and effective interaction. The roles of human-robot trust and technology affinity as mediators in the relationship between robot usability and worker willingness were analyzed in this study. As other critical variables, the mediator roles of Age and STARA were also calculated.

**Materials and methods:**

This study included 400 workers from a car company who interacted with industrial robots in their daily work activities. After examining the questionnaires’ validity and reliability, the main variables were determined to be willingness to use robots and robot usability. AMOS software also considered human-robot trust and worker technology affinity as mediators. The bootstrapping method was used to evaluate indirect relationships. A set of goodness-of-fit indices were presented to determine the adequacy of the goodness of fit between the proposed model and the data.

**Results:**

Based on model fit indices, an overall satisfactory model fit was obtained for the direct/indirect relationship between robot usability and worker willingness to use it (with mediating role of human-robot trust). Workers’ age and fear of Smart Technology, Artificial Intelligence, Robotics, and Algorithms (STARA) were identified as moderators in the relationship between usability and willingness.

**Conclusion:**

Attention to the robot usability and the role of workers’ trust in robots appears to be required to ensure workers’ willingness to use robots and the success of human-robot collaboration in future workplaces. As the workers age and their fear of robots grows, usability can play a larger role in increasing their willingness to put robots to work.

## Introduction

The recent revolution in workplaces and industries is referred to as Industry 4.0 and human–robot collaboration is the key component of this concept [1]. It is possible that in the near future, robots will enter factories alongside workers, implying that future workplaces will rely on worker–robot collaborations [2]. Workers must interact with industrial collaborative robots in this regard [1, 3]. Now that the need for robots in the workplaces has been established, businesses must prepare their workers for an effective and efficient interaction with robots [4]. The concept of worker 4.0 has also been introduced for this purpose [5]. Workers’ willingness to collaborate with industrial robots is a basic requirement for an efficient and effective interaction [6, 7]. The importance of workers’ willingness to interact with a robotic co-worker is a new topic which has been reported in a few studies [8]. Additionally, as with robots, willingness to use other emerging technologies can be used as an example [9–11]. Various parameters influence people’s willingness to interact with robots and choose robotic coworkers [12]. These parameters pertain to both the human and robotic components of the interaction [13]. Some of these parameters are more important than others. In the literature on human-robot interaction, the level of worker trust is extremely important [14, 15]. Sanders et al. found that workers’ trust in robots influences their willingness to choose whether or not to hire a robotic coworker [16]. Some aspects of the usability concept, such as a robot’s efficacy, are also regarded as critical parameters in the willingness to put robots to work [17]. Aside from these robot-based parameters, some studies have suggested that human factors such as personal emotion play a role in the willingness concept [6]. However, some other human-based parameters, such as the worker’s “technology affinity”, have yet to be studied. As an important human-based parameter in a worker-industrial robot collaboration, technology affinity appears to mean that a worker has more information and technical expertise about the complexity of technology than a robot [18]. As a result, an affinity technology worker differs from other workers in terms of their willingness to use industrial robots.

To the best of our knowledge, no study has been found that models a worker’s willingness to use an industrial robot in terms of its usability and the workers’ degree of trust.

The aim of this article was to shed light on the roles of technology affinity (Hypothesis 2) and human-robot trust (Hypothesis 3) as mediators, as well as a worker’s age (Hypothesis 4) and fear of robots (Hypothesis 5) as moderators of the association between robot usability and willingness to use it.

Hypothesis 1: There is a direct relationship between robot usability and the willingness to use the robot.
Hypothesis 2: The degree of a worker’s technological affinity can be used as a mediator in the relationship between a robot’s usability and willingness to use it.
Hypothesis 3: The degree of trust a worker has in robots can act as a moderator in the relationship between usability and willingness to use robots.
Hypothesis 4: The age of the worker can play a moderating role in the relationship between robot usability and willingness to use it.
Hypothesis 5: The fear of workers being replaced by robots can act as a moderator in the relationship between robot usability and willingness to use that robot.

## Materials and methods

This research was divided into two parts. The validity and reliability of the questionnaires were determined in the first section. The conceptual model of the study was tested in the second phase at one of the largest car companies in the Middle East.

### Ethical consideration

This study was approved (IR.UMSHA.REC.1399.133) by the ethical review board of the Hamadan University of Medical Sciences, Iran.

### Participants

The current study’s first phase included lay experts from the car company and academic experts with expertise in ergonomics and human-robot interaction (N = 12). In the second phase, all workers from the car company’s body unit who were directly involved in their work with the robots were invited to participate in the study. Industrial robots were used in this unit to perform press-related tasks as well as precise welding. Workers work in tandem with these robots to program, debug, and monitor the entire process. All of these employees were men who worked in shifts (N = 400).

### The original version of instruments

#### Willingness to use a robot

The workers’ willingness to use a robot was examined using the Persian version of the "service robot integration willingness scale” [12]. The 36 items in this questionnaire are divided into six subscales: performance efficacy, intrinsic motivation, anthropomorphism, social influence, facilitating condition, and emotions. The items in the Persian version were changed to improve the questionnaire’s applicability in an industrial setting. As a result of an extensive validity and reliability analysis, some subscales such as anthropomorphism and social influence were removed from the Persian version of the questionnaire. These questions were removed based on the research team’s decision and due to the lack of application of these dimensions in human-robot interaction in industrial settings.

#### Usability

The system usability scale is a technology-independent and dependable tool for assessing usability [17]. This 10-item questionnaire is thought to be reliable and valid for measuring users’ perceived usability in human-robot interaction [17, 19]. This instrument is used to rate the usability of human-robot interactions on a scale of 0 (poor) to 100 (good).

#### Technology affinity

Because perceived usability ratings are expected to vary depending on the degree of affinity for technology, this concept was evaluated using the affinity for technology interaction scale [20]. The original version of the instrument has 9 items. All of the questions were rated on a 6-point Likert scale ranging from strongly disagree to strongly agree.

#### Human-robot trust

Trust has been identified as a critical factor in the maintenance of human-robot relationships [14]. This concept was evaluated based on a study conducted by Peter A. Hancock and K Schaefer at Central Florida University [21]. In accordance with the recommended guidelines, we used the 14-item version of the instrument in the current study rather than the 40-item version.

#### STARA

Smart Technology, Artificial Intelligence, Robotics, and Algorithms are all abbreviations for this term. This four-item survey assessed employees’ perceptions of our future workplace. We can use this instrument to determine how much employees believe their jobs could be replaced by these types of technologies.

*Translation procedure*. The linguistic validation technique was used, as recommended by the literature. Two bilingual expert translators worked on the translation from English to Persian. The researchers and the translators then reached a consensus on the Persian version of the questionnaires. The Persian version was then translated back into English by one translator with an academic background in human-robot interaction and ergonomics who was unaware of the original version. The main authors endorsed the content of the English version produced by the research team in several correspondences. The purpose of this step was to ensure that the content was identical to the original. The original and back-translated versions were checked item by item with the help of translators and primary researchers. The final Persian versions of questionnaires were prepared as a result of these meetings.

*Validity and reliability of the questionnaires*. The Lawshi’s method for analyzing Content Validity Ratio (CVR) was used in this study to assess the validity of the questionnaires. The experts were given the questionnaires that had been prepared. The responses were divided into three categories: "Necessary," "Useful but Unnecessary," and "Unnecessary." Based on the completed questionnaires, the CVR was calculated:

$$\text{CVR}=\frac{ne-\frac{N}{2}}{\frac{N}{2}}$$

ne: number of persons responding to requested questions
N: total number of experts

Following the determination of the CVR, the Content Validity Index (CVI) and Scale-level Content Validity Index/ Averaging Calculation Method (S-CVI/AVE) were calculated to ensure that the best questions were selected for the questionnaire.

Determining the Cronbach’s α is an accepted method for evaluating the reliability and internal consistency of the Likert-based questionnaires. In the current study, this method was used to test the reliability of the proposed questionnaire. The α should be at least 0.7 to remain in the assessment tool. The obtained score of 0.7 indicates 70 percent stability for the proposed tool’s calculated scores (questionnaire).

*Data gathering procedure*. In the second phase, the corresponding author of the current study was present in the workplace. He distributed the questionnaires to the workers after explaining the objectives and receiving written consent to participate in the study. Attempts were made to complete the questionnaires without interfering with the workers’ work assignments, and to ensure that they had enough time to study and answer the questions thoroughly.

**Fig 1:**
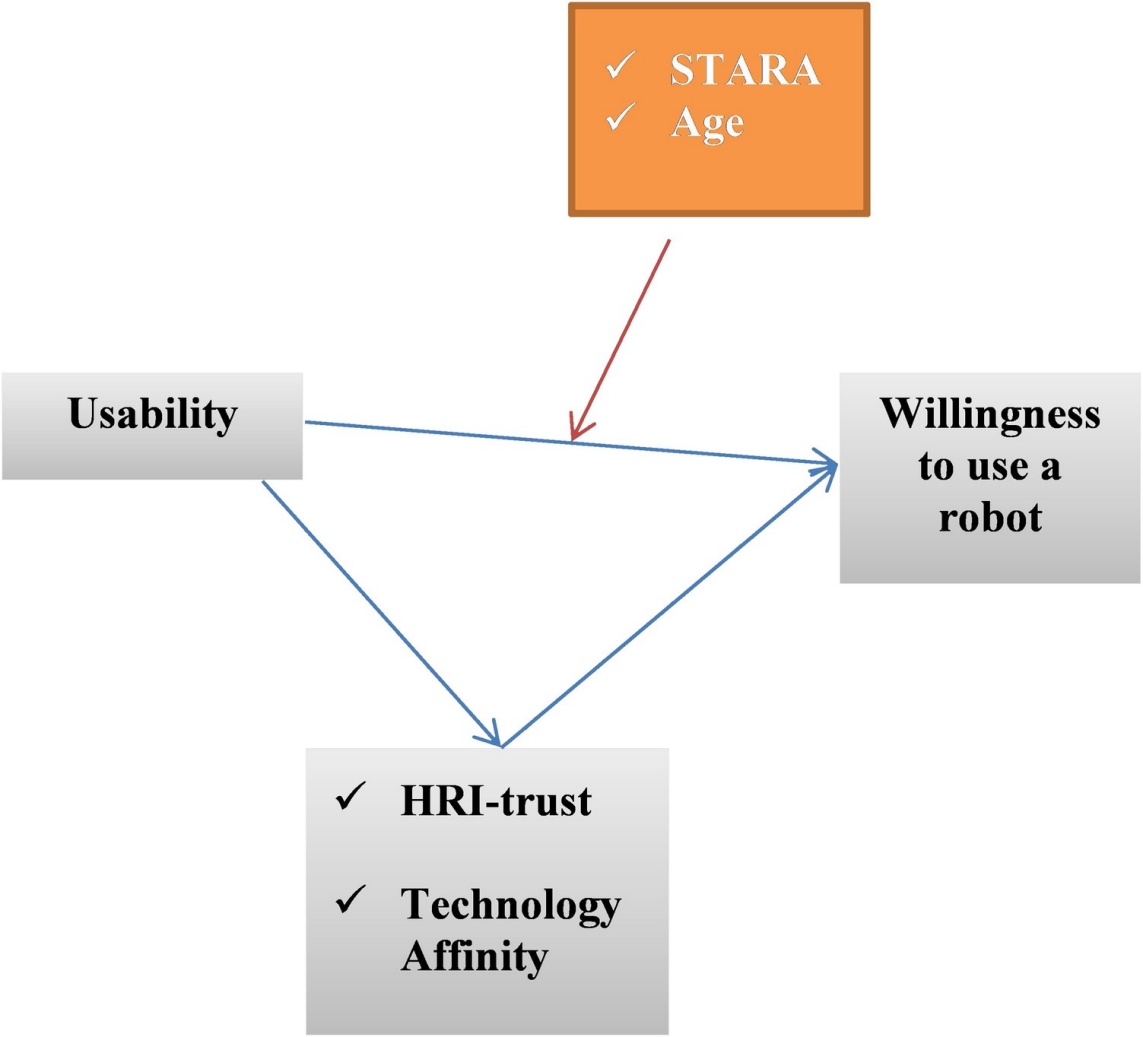
The conceptual model of the current study.

*Model specification*. The usability of robots as an independent latent variable and willingness to use a robot as a dependent variable comprise our proposed conceptual model. We hypothesized, first, that a high usability score is associated with workers’ willingness to use the robot. Second, we hypothesized that this association is mediated by personal technology affinity and trust in robots (Fig 1). The third hypothesis of the current study was the moderator effect of worker age and STARA in the previously mentioned model.

*Data analysis*. To summarize the demographic information, willingness to use robot, and usability scores, descriptive statistics were used. AMOS version 18 was used to conduct a path analysis to investigate the relationship between a robot’s usability and willingness to use it, including a potential mediating role of personal technology affinity and trust in robot, as well as a mediator role of Age and STARA. The raw data were exported from SPSS version 21 as a prerequisite for using structural equation modeling (SEM).

The research design was of the correlational type, with path analysis as a type of multiple regression analysis. The AMOS software was also used to assess the goodness of fit of the proposed model (Fig 1). The proposed model’s indirect effects (interfaces) were similarly investigated using a bootstrapping method in the Macro program. A set of goodness-of-fit indices were used to determine the adequacy of the goodness of fit between the proposed model and the data:

a) Chi-square value (X^2^), b) normalized index for Chi-square (X^2^/df), c) goodness of fit index (GFI), d) normalized fitness index (NFI), e) comparative fitness index (CFI) and f) root mean square error of approximation (RMSEA).

The indirect effect was considered significant if the 95-percent confidence interval (CI) excluded zero. A two-tailed p-value < 0.05 was deemed significant.

## Results

Table 1 summarizes the workers’ socio-demographic and job characteristics. The participants in this study have an average age of 38.7 years and 14.6 years of work experience.

**Table 1:** Selected socio-demographic, and job characteristics of the participants.

Variable	n/mean	% / SD
**Age**	38.7	5.04
**Work experience**	14.6	4.8
**Marital status**		
Single	38	10
Married	341	90
**Education level**		
Lower than BSc	311	81.6
BSc	49	12.9
MSc or higher	21	5.5

**Fig 2:**
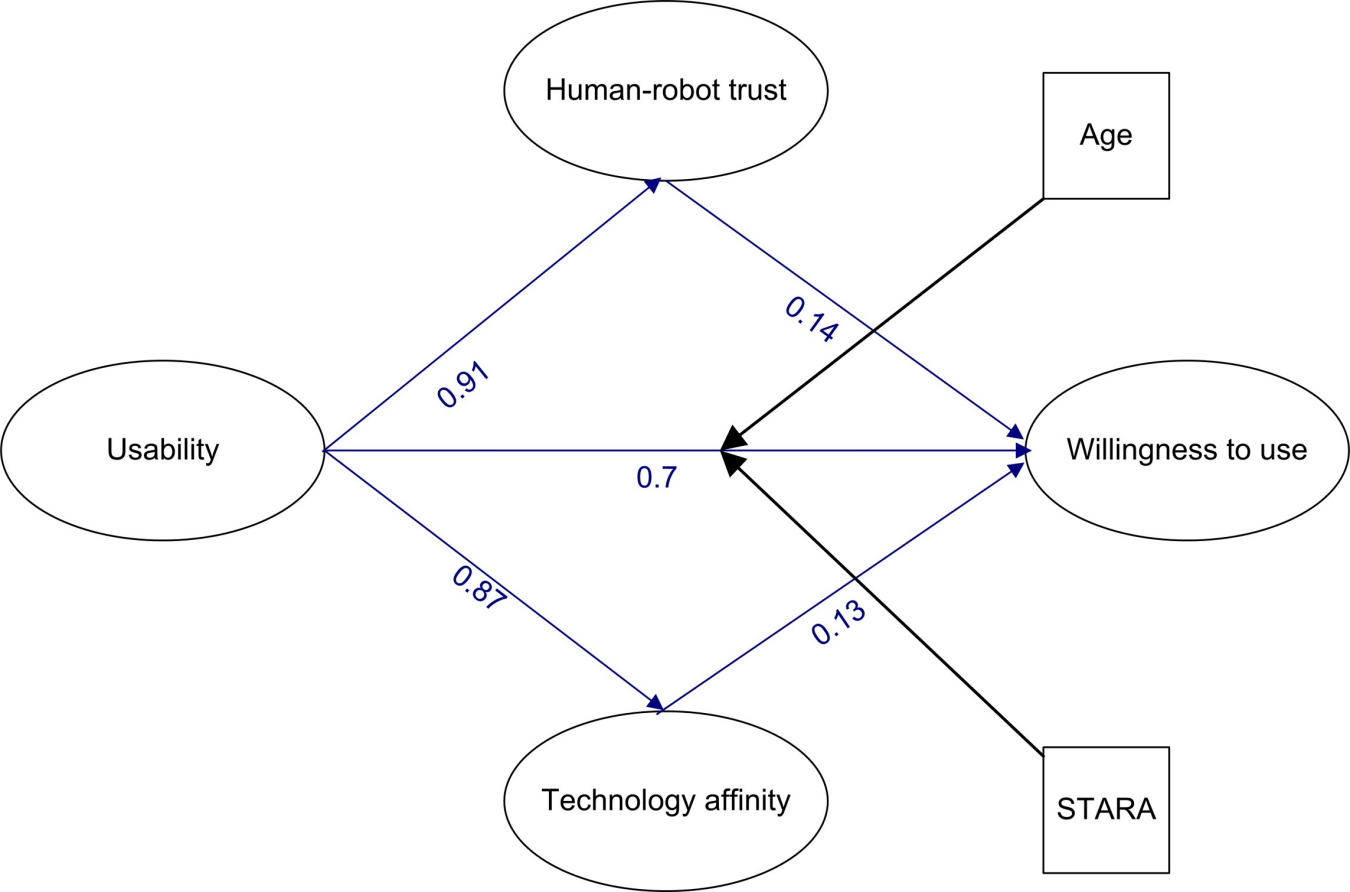
Final model of the present study along with its standardized path coefficients. ($x^{2}$: 6655.59; Df: 1409; x2df: 4.72; GFI: 0.57; AGFI: 0.53; IFI: 0.80; TLI: 0.79; CFI: 0.80; NFI: 0.76; RMSEA: 0.09).

Fitness indices between the data and the suggested model were calculated. These results are depicted in Fig 2. Based on model fit indices, an overall satisfactory model fit was achieved.

The model’s standardized estimates are also shown in Fig 2 and Table 2. Table 3 shows that all of the standardized path coefficients in the proposed model were significant. As a result, direct relationships were also permitted.

**Table 2:** Direct effects in the present study.

Path			Estimate	p
**trust**	<---	usability	.907	0.001
**affinity**	<---	usability	.867	0.001
**willingness**	<---	trust	.143	0.016
**willingness**	<---	affinity	.133	0.006
**willingness**	<---	usability	.705	0.001

**Table 3:** Results of bootstrapping.

Indirect path		Data	Boot	Bias	Standard error	Lower bound	Upper bound
**A**	**Usability→ Human-robot trust →Willingness to use**	0.069	0.072	0.003	0.033	0.013	0.144
**B**	**Usability→ technology affinity →Willingness to use**	0.024	0.024	0.000	0.020	-0.002	0.077

Table 3 displays the results of the mediation analysis using the bootstrapping method. According to this, confidence intervals for the paths drawn in the table showed no zero points with respect to the indirect path A, but there is a zero point in the indirect path B, confirming the indirect relationship of usability with willingness via human-robot trust. As a result, hypothesis 2 is not supported, whereas hypothesis 3 is supported in the current study.

The confidence level associated with this interval was 95, and the number of bootstrapping re-samplings was 1000.

The method presented by Hayes et al. was used to analyze the moderating effects of age and STARA on the relationship between usability and willingness [22]. According to the findings (Table 4), age and STARA both have a moderating effect on the relationship between usability and willingness to use. As a result, the current study supports both hypothesis 4 and hypothesis 5.

**Table 4:** Results of moderation analysis.

Moderating effect	R2-chng	F	df1	df2	p
**Age as moderator**	0.009	4.291	1	3751	**0.039**
**STARA as moderator**	0.013	6.269	1	330	**0.012**

**Fig 3:**
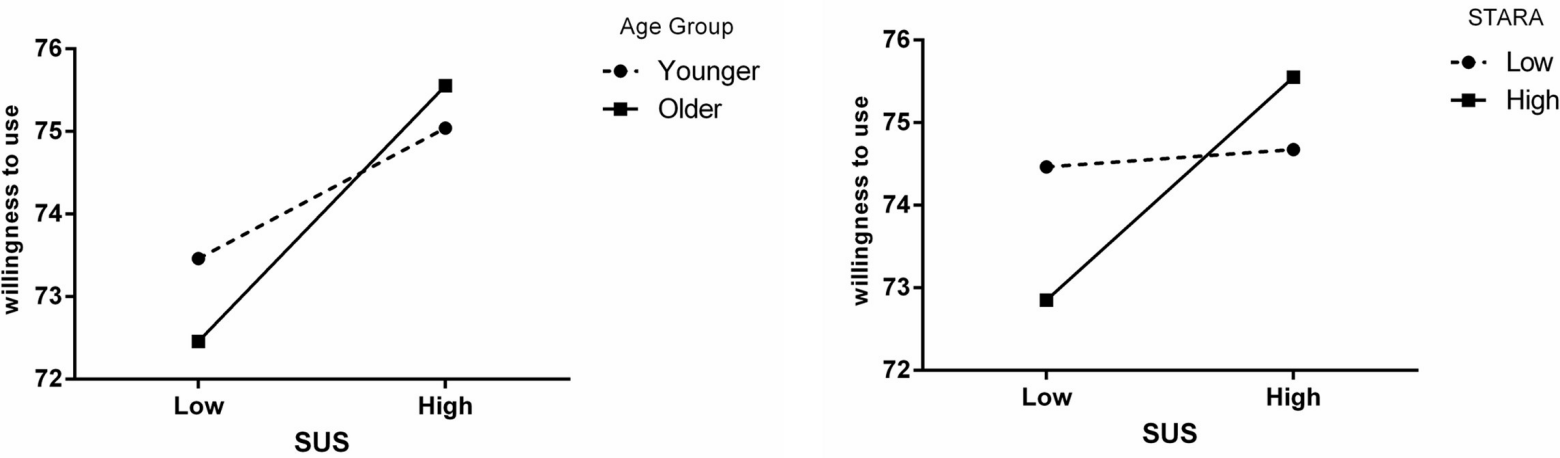
Moderating effect of age and STARA on the relationship between usability of a robot and willingness to use it.

As shown in Fig 3, both variables play a significant moderating role in the relationship between the independent and dependent variables. This graph shows that in people with higher levels of fear, the higher the usability of a robot, the greater the likelihood of success in using that robot. As a result, it is preferable to pay more attention to robot usability in order to increase the chances of successful human-robot cooperation in future work environments.

This figure also shows that the older the users are, the more likely it is that they will use a robot. As a result, as the population of most countries ages, one of the keys to industry’s success in the Fourth Industrial Revolution will be to pay attention to the usability of equipment, technologies, and robots.

## Discussion

Nobody can deny the significance of using intelligent technologies based on artificial intelligence, such as robots, and their impact on increasing the efficiency and effectiveness of manufacturing processes [23, 24].

The purpose of this study was to determine the role of human-robot trust and workers’ technology affinity in mediating the relationship between robot usability and willingness to use it.

The current study approved the direct relationship between a robot’s usability and workers’ willingness to use that robot.

According to the findings of a study conducted by Plaza et al., as a product’s user friendliness and usability improve, so does the willingness to use that technology [25]. In line with this finding, a study of French participants concluded that a robot’s usability design can increase its chances of being accepted by users [26]. Acceptance or willingness to use a robot was linked to some factors such as its usability in a systematic review [27]. Regardless of the context in which a robot is used, it seems logical to conclude that the more usable a robot is, the more likely it is to be accepted by end users and the more willing they are to use it.

In this study, we proposed that trusting a robot mediates the relationship between its usability and willingness to use it. According to the results, this path was approved.

We proposed this path for the first time, and due to limitations in the literature, we presented other researchers’ opinions about the two direct relationships that formed our proposed path for discussion. The first was a direct relationship between usability and trust, while the second was a relationship between trusting a robot and willingness to use it.

Separately, usability, trust, and willingness have been widely discussed in the field of human-robot interaction [6, 7, 17, 28]. In recent years, there has been an increase in the belief that the higher the level of usability of a robot, the greater the level of trust in that robot [29]. Furthermore, some studies have confirmed that one of the important factors ensuring the use of a new technology is the level of trust in that technology [16, 30, 31]. Other studies have shown that trust and usability are two important factors in accepting and implementing new technologies [32]. Despite the fact that only a few studies have examined the effect of trust on willingness to use a new technology [33], it can be concluded that trust is one of the important pathways between usability and willingness to use intelligent technology.

Despite the effect of robot usability parameters on user technology affinity characteristics, other findings of the current study did not support the role of worker technology affinity characteristics as a mediator in the relationship between robot usability and willingness to use it.

Prior research has supported the effects of technology affinity on the behavior of technological product users, and some researchers argue that understanding individual differences in terms of technological affinity could be useful for the design and use of high-tech products [34–36]. The industrial context of the current study may be one of the reasons for rejecting the mediating role of technology affinity in this regard. The technology affinity construct is typically applied in non-industrial contexts such as driving (semi) autonomous vehicles or using mobile phones [20]. Another factor that can be cited to support the current findings is the concept of technology affinity itself. This term refers to a personal characteristic. Indeed, it can be argued that a person with this feature, regardless of a product’s usability, can fall in love with it because of its inner desire and does not see its flaws or underestimate it.

In another part of the study, we looked at how age and STARA affected the relationship between industrial robot usability and willingness to use it. Both the workers’ age and STARA can act as moderators. It means that at different levels of these two moderating variables, there is a significant difference in the relationship between the two independent and dependent variables.

According to the findings, the older the users are, the greater the usability of a robot can increase the likelihood of using it. Several variables have been used as moderators in previous studies to study user acceptance of technology. Aboobucker et al. investigated the role of age as a moderator in the relationship between the usability of an internet banking website and users’ acceptance and willingness to use that technology [32]. Furthermore, age was investigated as a moderator between perceived usefulness and intention to use a mobile application in a recent study [37]. As a result, age of users could not moderate the main relationship, which contradicts the findings of the current study. The difference between the two studies could be attributed to the differences in the studied technologies (website/application vs. industrial robot). In other words, as technology becomes easier to use, the moderating role of age diminishes.

From another angle, it can be argued that the age pyramid of countries will result in more old workers in future workplaces [38, 39]. As a result of the age-moderating role findings, more emphasis should be placed on the usability of robots in future work environments to ensure the success of the older worker-robot collaboration.

Fear of losing a job due to technological replacement, as measured by the STARA concept, was tested as the other moderating variable. The higher the usability of a robot in people with higher fear, the higher the willingness to use that robot. As a result, it is preferable to prioritize robot usability in order to increase the chances of successful human-robot collaboration in future work environments.

In general, if a person is concerned about being replaced by intelligent technology in the future, it is recommended that people be influenced through psychological interventions to reduce their inner fear and increase the chances of success of human cooperation with robots in future work environments.

When interpreting the findings, some limitations of this study should be considered. This study was conducted in the form of a cross-sectional study. Longitudinal studies can be conducted to further validate the findings of the cross-sectional analysis used in this study. For generalizability, such a study should be repeated in other cultures to account for potential cross-cultural differences.

## Conclusion

In order to increase workers’ willingness to use robots, other parameters such as usability should be considered in addition to robot technical and safety parameters. The usability of robots, either directly or indirectly by increasing worker trust in robots, can ensure effective collaboration between humans and robots. Furthermore, in this age of aging, consideration should be given to parameters such as worker age. Fear of being replaced by robots is another important factor to consider when designing a human-robot interaction.
